# Kill two birds with one stone: one submucosal tunnel for achalasia combined with large epiphrenic diverticulum

**DOI:** 10.1055/a-2163-1924

**Published:** 2023-09-21

**Authors:** Jingjing Lian, Aiping Xu, Tao Chen, Meidong Xu

**Affiliations:** Endoscopy Center, Department of Gastroenterology, Shanghai East Hospital, Tongji University School of Medicine, Shanghai, China

Esophageal epiphrenic diverticulum (EED) in the distal 10 cm of the esophagus usually occurs in patients with esophageal motility disorders including achalasia. Per-oral endoscopic myotomy (POEM) is an effective and minimally invasive method that has developed into an important treatment for achalasia 1 . In 2016, we created submucosal tunneling endoscopic septum division (STESD) as a novel technique for treating Zenker’s diverticulum 2 . Here we present a case of achalasia with a large epiphrenic diverticulum that we cured with POEM and STESD together using only one submucosal tunnel.

A 55-year-old man was referred to our center with complaints of severe dysphagia, regurgitation, and significant weight loss. Gastroscopy, high-resolution manometry, a computed tomography (CT) scan, and barium swallow were examined and revealed achalasia cardia with a large esophageal epiphrenic diverticulum ( Fig. 1 ). The procedure was performed as follows ( Video 1 ) and is very similar to the one reported by Inoue et al 3 . First, a submucosal injection and mucosal incision were made at the posterior wall, 13 cm above the esophagogastric junction. Then a submucosal tunnel was created from the mucosal incision, distally extending about 3 cm into the cardia. When passing the diverticulum, the tunnel was fully expanded to sufficiently expose the septum ( Fig. 2 ). Next, the muscle of the diverticulum septum was completely cut off to widely open the diverticulum. Then myotomy was performed from the proximal end of the diverticulum to 3 cm below the tunnel entry and distally stretched into the end of the tunnel. Finally, the mucosal entry site was closed. The patient recovered very well and was discharged on the third postoperative day. After 6 months, he had gained about 10 kg and all the symptoms were completely resolved. Gastroscopy confirmed the disappearance of the large diverticulum and a smooth passage of the cardia site ( Fig. 3 ).

**Fig. 1:**
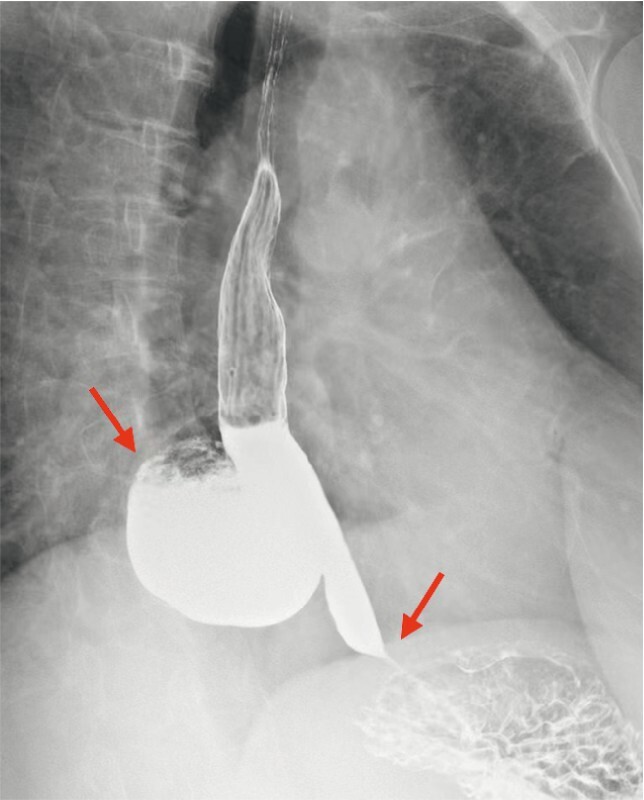
Contrast swallow showed a large epiphrenic diverticulum in the lower esophagus.

**Video 1** Peroral endoscopic myotomy combined with submucosal tunneling endoscopic septum division using only one submucosal tunnel.


**Fig. 2:**
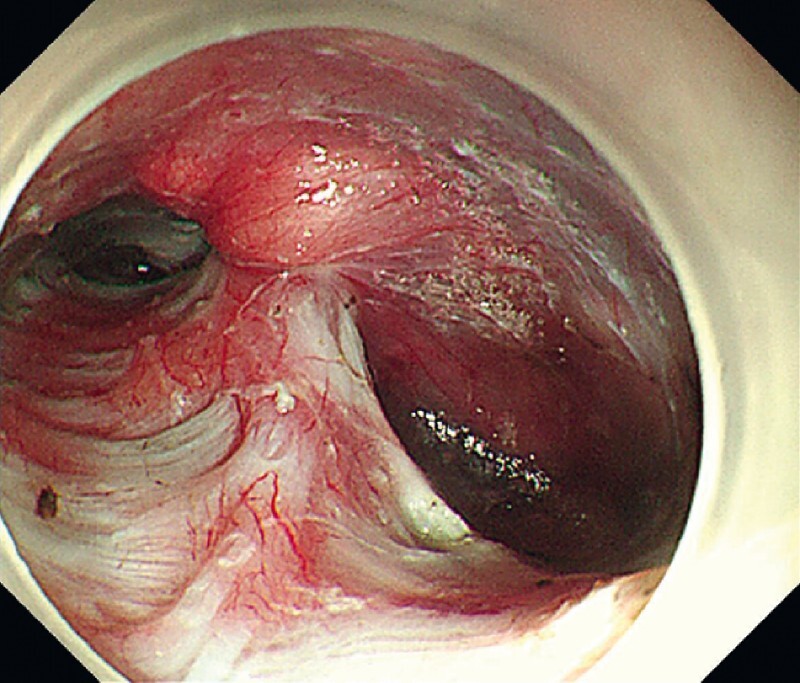
Endoscopic view of the diverticulum within the submucosal tunnel.

**Fig. 3:**
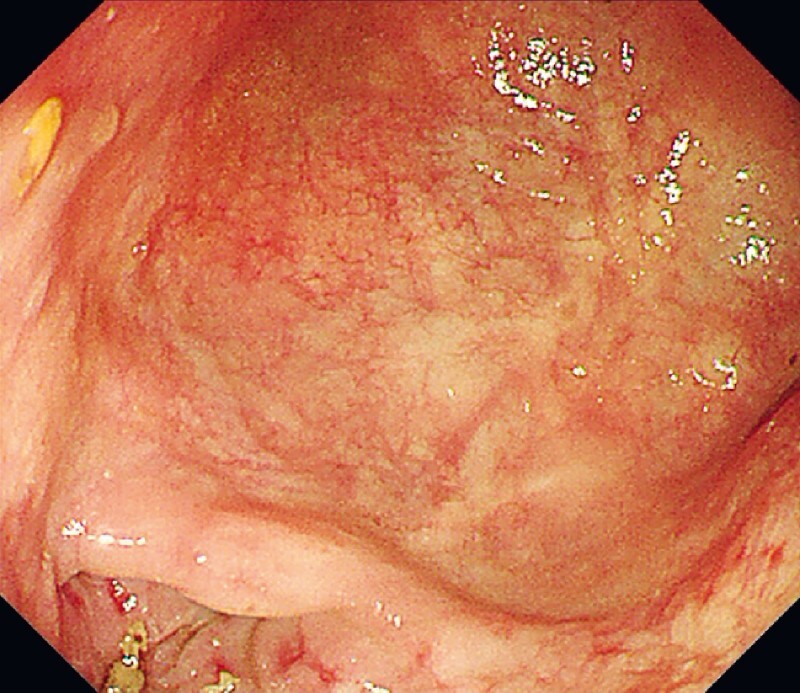
Endoscopic view of the collapsed septum at the diverticulum site after 6 months.

Endoscopy_UCTN_Code_TTT_1AO_2AN

## Notes

Endoscopy E-Videos

https://eref.thieme.de/e-videos
*E-Videos* is an open access online section of the journal *Endoscopy* , reporting on interesting cases and new techniques in gastroenterological endoscopy. All papers include a high-quality video and are published with a Creative Commons CC-BY license. Endoscopy E-Videos qualify for HINARI discounts and waivers and eligibility is automatically checked during the submission process. We grant 100% waivers to articles whose corresponding authors are based in Group A countries and 50% waivers to those who are based in Group B countries as classified by Research4Life (see: https://www.research4life.org/access/eligibility/ ). This section has its own submission website at https://mc.manuscriptcentral.com/e-videos
